# Influence of Rice–Crayfish Co-Culture Systems on Soil Properties and Microbial Communities in Paddy Fields

**DOI:** 10.3390/plants14152320

**Published:** 2025-07-27

**Authors:** Dingyu Duan, Dingxuan He, Liangjie Zhao, Chenxi Tan, Donghui Yang, Wende Yan, Guangjun Wang, Xiaoyong Chen

**Affiliations:** 1 College of Ecology and Environmental, Central South University of Forestry and Technology, Changsha 410004, China; 2019210003@xyafu.edu.cn (D.D.); t20001421@csuft.edu.cn (W.Y.); 2 College of Fisheries, Xinyang Agriculture and Forestry University, Xinyang 464000, China; a850924t@163.com (L.Z.); tanchenxi2013@163.com (C.T.); 1990210012@xyafu.edu.cn (D.Y.); 3 College of Pharmacy, Xinyang Agriculture and Forestry University, Xinyang 464000, China; hdxmusic@xyafu.edu.cn; 4 National Engineering Laboratory for Applied Technology of Forestry and Ecology in South China, Changsha 410004, China; 5 College of Arts and Sciences, Governors State University, University Park, IL 60484, USA

**Keywords:** integrated rice–crayfish system, soil properties, microbial communities, nutrient cycling, paddy fields, sustainable agriculture

## Abstract

Integrated rice–crayfish (*Oryza sativa*–*Procambarus clarkii*) co-culture (RC) systems have gained prominence due to their economic benefits and ecological sustainability; however, the interactions between soil properties and microbial communities in such systems remain poorly understood. This study evaluated the effects of the RC systems on soil physicochemical characteristics and microbial dynamics in paddy fields of southern Henan Province, China, over the 2023 growing season and subsequent fallow period. Using a randomized complete design, rice monoculture (RM, as the control) and RC treatments were compared across replicated plots. Soil and water samples were collected post-harvest and pre-transplanting to assess soil properties, extracellular enzyme activity, and microbial community structure. Results showed that RC significantly enhanced soil moisture by up to 30.2%, increased soil porosity by 9.6%, and nearly tripled soil organic carbon compared to RM. The RC system consistently elevated nitrogen (N), phosphorus (P), and potassium (K) throughout both the rice growth and fallow stages, indicating improved nutrient availability and retention. Elevated extracellular enzyme activities linked to carbon, N, and P cycling were observed under RC, with enzymatic stoichiometry revealing increased microbial nutrient limitation intensity and a shift toward P limitation. Microbial community composition was significantly altered under RC, showing increased biomass, a higher fungi-to-bacteria ratio, and greater relative abundance of Gram-positive bacteria, reflecting enhanced soil biodiversity and ecosystem resilience. Further analyses using the Mantel test and Random Forest identified extracellular enzyme activities, PLFAs, soil moisture, and bulk density as major factors shaping microbial communities. Redundancy analysis (RDA) confirmed that total potassium (TK), vector length (VL), soil pH, and total nitrogen (TN) were the strongest environmental predictors of microbial variation, jointly explaining 74.57% of the total variation. Our findings indicated that RC improves soil physicochemical conditions and microbial function, thereby supporting sustainable nutrient cycling and offering a promising, environmentally sound strategy for enhancing productivity and soil health in rice-based agro-ecosystems.

## 1. Introduction

The integrated rice–crayfish (*Oryza sativa–Procambarus clarkii*) co-culture (RC) has gained increasing attention in recent years due to its economic benefits, ecological sustainability, and contribution to food security [1,2]. Widely practiced in southern China and other Asian countries, this RC system not only improves land-use efficiency but also enhances soil nutrient recycling and reduces the need for chemical fertilizers and pesticides [3]. RC represents a complex agro-ecosystem where biotic and abiotic factors interact dynamically. Unlike rice monoculture (RM), the introduction of crayfish brings unique bioturbation effects and organic matter redistribution, which can alter microhabitats for microbial colonization and activity. Such interactions may enhance the functional diversity and redundancy of microbial communities, potentially stabilizing ecosystem processes under environmental fluctuations [4]. Furthermore, the reduced reliance on chemical inputs in co-culture systems aligns with global sustainable agriculture goals by mitigating adverse environmental impacts and fostering soil health restoration. However, despite these benefits, the biogeochemical interactions occurring within RC, particularly between soil properties and microbial communities, remain inadequately understood.

The rice–crayfish (*Oryza sativa–Procambarus clarkii*) co-culture system (RC) has emerged as an innovative and ecologically beneficial model of integrated agriculture, especially in China’s Yangtze River Basin. This system combines rice farming with freshwater crayfish cultivation in the same paddy field, offering synergistic benefits such as enhanced nutrient cycling, improved soil structure, pest and weed suppression, and increased land-use efficiency [5,6]. Crayfish bioturbation promotes aeration and nutrient redistribution, while their excretions enrich the soil with organic matter and available nutrients, supporting rice growth [7]. Moreover, the RC model reduces the need for chemical fertilizers and pesticides, aligning with sustainable agriculture goals. Due to its economic and ecological advantages, the RC system has been rapidly adopted in southern China and is now recognized as a key strategy for green development in rural areas [8]. However, its impacts on soil biochemical processes and microbial communities remain underexplored, highlighting the need for systematic research to understand the underlying mechanisms and guide optimized management.

Soil microbial communities are central to nutrient cycling, organic matter decomposition, and the transformation of nitrogen (N) compounds [9]. In rice-based agro-ecosystems, microbial activity is strongly influenced by flooding regimes, organic inputs, and animal activity, all of which are modulated in RC systems [10]. The burrowing and foraging behavior of crayfish alters soil physical structure, redox potential, and nutrient dynamics, potentially influencing microbial composition and functional pathways [11]. Soil properties such as pH, total nitrogen (TN), available phosphorus (AP), and soil organic carbon (SOC) are critical regulators of microbial community structure [12,13]. Changes in these properties, induced by the presence of aquatic animals in paddy fields, may shift microbial assemblages and affect ecosystem functions such as nitrification, denitrification, and organic matter mineralization [14]. Therefore, evaluating the interactions between soil physicochemical characteristics and microbial communities in RC is essential to understand their impact on soil fertility, sustainability, and crop productivity. However, few studies have systematically assessed how RC influences both soil properties and microbial dynamics across growing seasons. Seasonal variations driven by fluctuations in water management, temperature, and plant growth stages can profoundly affect microbial community structure and function. However, these dynamics remain insufficiently characterized in RC systems. A comprehensive understanding of these temporal patterns is essential for developing management practices that enhance both productivity and ecosystem resilience.

This study aims to investigate the effects of the RC system on soil physicochemical properties and microbial community composition compared to a traditional RM system. We hypothesize that the RC system significantly alters soil physicochemical characteristics and microbial communities, resulting in enhanced nutrient cycling and improved soil fertility throughout both the rice growing season and the fallow period. The objectives of this study were the following: (1) To quantify changes in soil physicochemical parameters and nutrient dynamics under RC relative to RM; (2) to characterize shifts in soil microbial community structure and extracellular enzyme activities across key growth stages; and (3) to elucidate the relationships between soil properties and microbial dynamics to inform sustainable management practices for RC systems. By addressing these objectives, this research aims to generate novel insights into soil–microbe interactions that underpin the sustainability of integrated aquaculture–agriculture systems, thereby providing a scientific foundation for optimizing co-culture practices that balance agricultural productivity with environmental stewardship.

## 2. Results

### 2.1. Effects of Rice–Crayfish Co-Culture Systems on Soil Physicochemical Properties

Two-way ANOVA revealed that both growth stage and treatment had highly significant effects (*p* < 0.001) on soil moisture content (MC), soil porosity (SP), bulk density (BD), and soil organic carbon (SOC). Treatment effects were also significant for pH (*p* < 0.001). Significant interaction effects (S × T) were observed for soil MC, pH, and SOC, indicating that the co-culture system’s impact on these parameters varied with plant developmental stage. As shown in Table 1, the RC system significantly altered soil physicochemical properties compared to RM across different growth stages. At the tillering stage, RC significantly increased soil MC, SP, and SOC by 30.2%, 9.6%, and 95.8%, respectively, while significantly reducing BD compared to RM (*p* < 0.001). No significant difference in soil pH was observed. During the jointing stage, RC plots exhibited higher soil MC, SP, and SOC (*p* < 0.001), while BD and pH were significantly lower than in RM (*p* < 0.001 for both). Notably, SOC in RC was nearly three times higher than in RM. At the heading stage, RC treatment again led to significantly higher MC, SP, and SOC, with SOC increasing more than threefold compared to RM (*p* < 0.001). BD was slightly lower under RC but not statistically different, whereas pH was significantly reduced. By the mature stage, the RC system maintained significantly higher MC, SP, and SOC (*p* < 0.001) while reducing BD and pH compared to RM. SOC in RC remained nearly three times that in RM. In the fallow stage, although both systems showed overall lower MC and SOC compared to the cropping stages, RC still had significantly higher MC, SP, and SOC and significantly lower BD than RM (*p* < 0.001). Soil pH differences were not statistically significant at this stage (Table 1).

### 2.2. Effects of Rice–Crayfish Co-Culture on Soil Nutrient Contents

**Figure 1:**
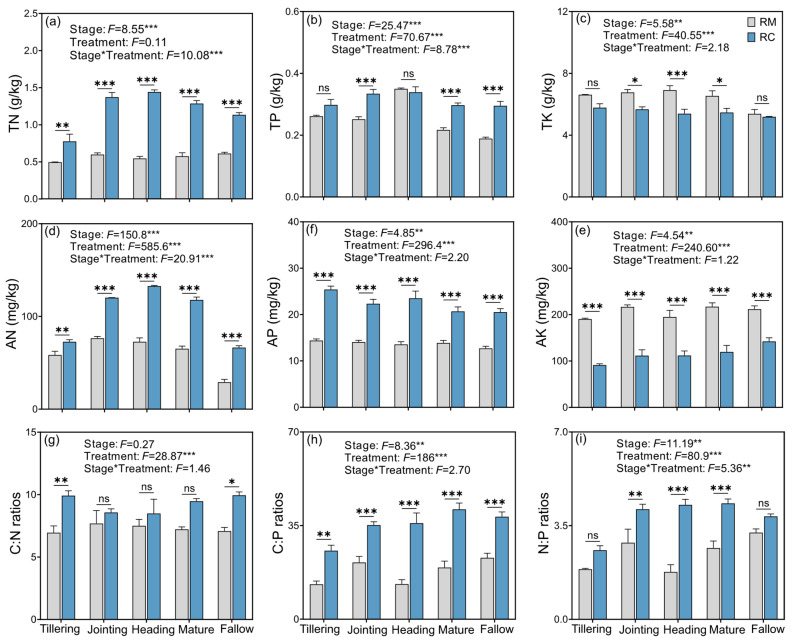
Effects of the rice–crayfish co-culture system on soil nutrient contents. (**a**–**i**) Changes in total nitrogen (TN), total phosphorus (TP), total potassium (TK), available nitrogen (AN), available phosphorus (AP), and available potassium (AK) under rice–crayfish co-culture (RC) and rice monoculture (RM) systems across five growth stages: tillering, jointing, heading, maturity, and fallow. Data are presented as mean ± SE (*n* = X). Asterisks indicate significant differences between treatments at each stage (* *p* < 0.05, ** *p* < 0.01, *** *p* < 0.001).

Figure 1a–e illustrates the dynamic changes in soil nutrient contents under the RC and RM systems across five stages: tillering, jointing, heading, mature, and fallow stages. At the tillering stage, TN, AN, and AP contents in the RC treatment were significantly higher than those in RM, with particularly pronounced increases in AN and AP. During the mid- to late stages of rice growth, soil nutrient levels in the RC system remained consistently higher than in RM. RC treatment significantly increased TN and AN contents. However, TK and AK contents significantly decreased under RC. TP and AP levels also remained elevated throughout the season. Even during the fallow period, TN, TP, AN, and AP contents in RC plots remained higher than in RM. In contrast, AK levels significantly declined under RC.

Figure 1g–i shows the changes in soil ecological stoichiometric ratios under the RC and RM systems across the same five stages. At the tillering stage, RC significantly increased the C:N and C:P ratios, while no significant difference was observed in the N:P ratio. In the mid- to late stages of rice growth, the C:P and N:P ratios increased significantly under RC, while the C:N ratio remained unchanged.

### 2.3. Effects of the Rice–Crayfish Co-Culture System on Soil Extracellular Enzyme Activities and Vector Characteristics

**Figure 2:**
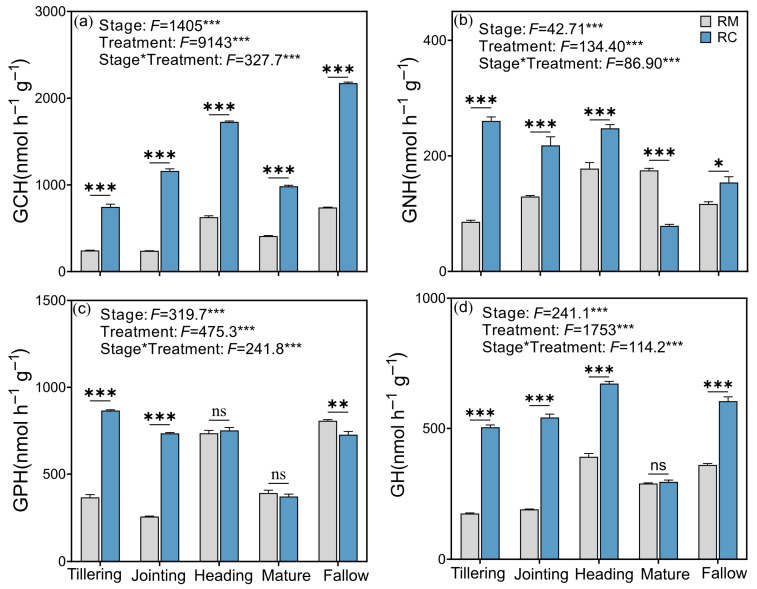
Effects of the rice–crayfish co-culture system on soil extracellular enzyme activities and microbial nutrient acquisition. (**a**) Geometric mean of carbon-hydrolyzing enzyme activities (GCH); (**b**) Geometric mean of nitrogen-hydrolyzing enzyme activities (GNH); (**c**) Acid phosphatase (ACP) activity related to phosphorus cycling; (**d**) Microbial nutrient acquisition vector characteristics. GH: geometric mean of five hydrolytic enzymes; GCH: geometric mean of C-hydrolyzing enzymes; GNH: geometric mean of N-hydrolyzing enzymes; ACP: acid phosphatase. Significance levels: *** *p* < 0.001; ** *p* < 0.01; * *p* < 0.05.

The RC system significantly enhanced soil microbial functional activity, particularly the activity of extracellular enzymes involved in nutrient cycling. Figure 2 illustrates the effects of RC and RM on the geometric mean activities of extracellular enzymes associated with C, N, and P cycling, as well as microbial nutrient acquisition strategies. Compared to RM, the RC system significantly increased the geometric mean activity of C-hydrolyzing enzymes (GCH; Figure 2a) at all growth stages, with highly significant differences (*p* < 0.001). The geometric mean activity of N-hydrolyzing enzymes (GNH; Figure 2b) was significantly elevated under RC at all stages except for the maturity stage, during which a significant decrease was observed. For P, the activity of acid phosphatase (ACP), a key enzyme involved in P mineralization, was significantly higher under RC during the early growth stages but showed no significant difference thereafter, followed by a marked decline during the fallow period (Figure 2c). Furthermore, the geometric mean of all five measured hydrolases (GH; Figure 2d) consistently remained higher under RC, although no significant difference was observed at the maturity stage.

**Figure 3:**
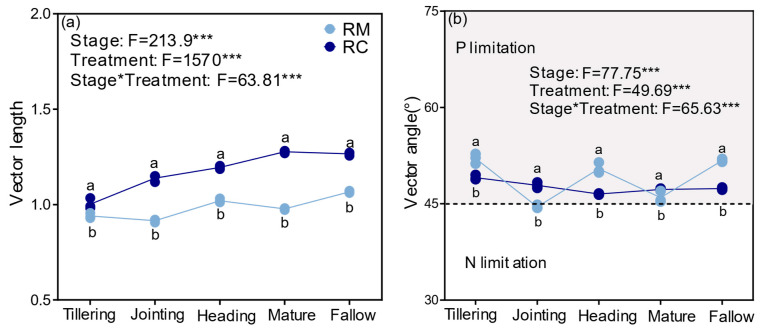
Effects of the rice–crayfish co-culture system on soil enzymatic stoichiometry: (**a**) vector length (VL), indicating the overall intensity of microbial nutrient limitation; and (**b**) vector angle (VA), where VA < 45° indicates nitrogen limitation and VA > 45° indicates phosphorus limitation. *** *p* < 0.001 indicates highly significant differences.

As shown in Figure 3a, vector length (VL), an indicator of overall microbial nutrient limitation intensity, was significantly greater under RC than RM throughout all growth stages. The significant interaction between stage and treatment (*p* < 0.001) confirms that the effect of RC on microbial nutrient demand varied with crop development. Figure 3b shows the vector angle (VA), which differentiates between N and P limitations (VA < 45° indicates N limitation; VA > 45° indicates P limitation). Across all stages, the RC system consistently exhibited VA values above 45°, indicating persistent microbial P limitation, whereas RM plots showed lower VA at the early stage (tillering), reflecting a temporary N limitation.

### 2.4. Effects of Rice–Crayfish Co-Culture System Models on Soil Microbial Phospholipid Fatty Acids (PLFAs)

**Figure 4:**
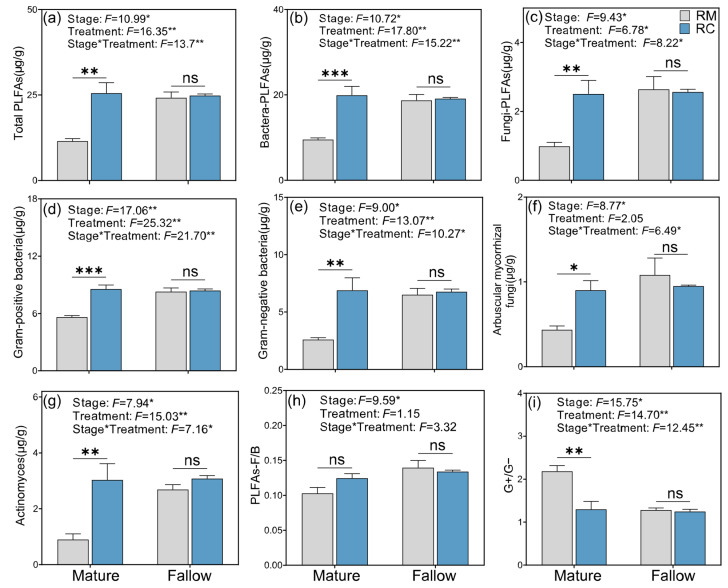
Effects of different rice–crayfish integrated farming systems on soil microbial community composition based on phospholipid fatty acid (PLFA) analysis, including (**a**–**c**) the changes in PLFA composition, (**d**) relative abundances of Gram-positive (G^+^) bacteria, (**e**) relative abundances of Gram-negative (G^−^) bacteria, (**f**) relative abundances of ACT, (**g**) relative abundances of AMF, (**h**) the ratio of fungal PLFAs (FPLFAs) to bacterial PLFAs (BPLFAs), and (**i**) the ratio of Gram-positive (G^+^) to Gram-negative (G^−^) bacterial PLFAs. Abbreviations are as follows: TPLFA, total phospholipid fatty acid content; BPLFA, bacterial phospholipid fatty acid content; FPLFA, fungal phospholipid fatty acid content; G^+^, Gram-positive bacteria; G^−^, Gram-negative bacteria; AMF, arbuscular mycorrhizal fungi; ACT, actinomycetes; F/B, ratio of fungal to bacterial PLFAs; and G^+^/G^−^, ratio of Gram-positive to Gram-negative bacterial PLFAs. Asterisks indicate the level of statistical significance: *** *p* < 0.001 (highly significant), ** *p* < 0.01 (very significant), and * *p* < 0.05 (significant).

At the maturity stage, soil microbial activity was higher, with phospholipid fatty acid (PLFA) content (indicated by blue bars) showing a marked increase compared to RM (indicated by gray bars) (Figure 4).

To further investigate microbial community structure, the ratio of fungal PLFA (FPLFA) to bacterial PLFA (BPLFA), as well as the ratio of Gram-positive (G^+^) to Gram-negative (G^−^) bacteria, was analyzed, as shown in Figure 4h,i. The results revealed that under RC, the F/B ratio showed no significant differences during the maturity and fallow stages. However, the G^+^/G^−^ ratio was significantly elevated at the maturity stage (*p* < 0.05), suggesting that Gram-positive bacteria became more dominant under these conditions.

### 2.5. Effects of Soil Environmental Factors on Microbial Community Composition Under Rice–Crayfish Co-Culture Systems

**Figure 5:**
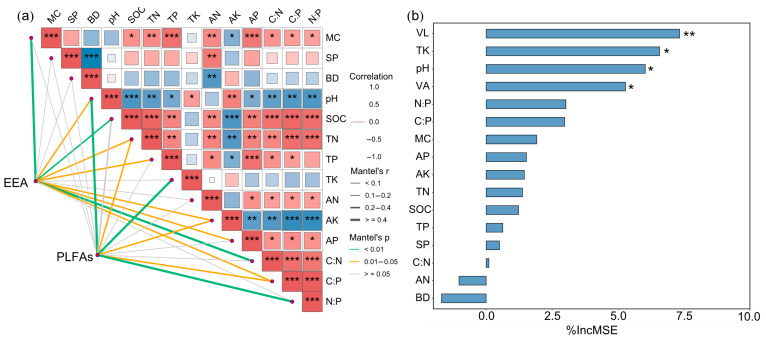
Results of the Mantel test (**a**) and Random Forest analysis (**b**) illustrating the effects of soil environmental factors on microbial community composition under different rice–crayfish integrated farming systems. Abbreviations: PLFAs, phospholipid fatty acids; EEA, extracellular enzyme activity; MC, soil moisture content; SP, soil porosity; BD, bulk density; SOC, soil organic carbon; TN, total nitrogen; TP, total phosphorus; TK, total potassium; AN, available nitrogen; AK, available potassium; AP, available phosphorus; VL, vector length; VA, vector angle. Asterisks indicate significance levels: *** *p* < 0.001 (highly significant), ** *p* < 0.01 (very significant), * *p* < 0.05 (significant).

The results demonstrated that both biochemical factors (e.g., PLFAs and extracellular enzyme activities [EEAs]) and physical properties (e.g., moisture content and bulk density) are key drivers influencing microbial community composition under RC systems (Figure 5). The Mantel test (Figure 5a) revealed significant correlations between microbial community structure and several soil environmental factors. Specifically, EEA was significantly correlated with MC, SOC, TN, TP, AK, AP, C:N, C:P, N:P ratios, and pH, while PLFAs were significantly correlated with TN, TK, AK, C:P, N:P, and pH (*p* < 0.01). Among these, MC, SOC, TN, TP, AN, AP, and stoichiometric ratios (C:N, C:P, N:P) showed significant positive correlations, whereas pH, BD, and AK were negatively correlated with microbial communities. PLFAs and EEA exhibited the highest correlation coefficients, highlighting their dominant roles in shaping microbial structure.

The Random Forest analysis (Figure 5b) further identified vector length (VL) as the most important predictor of microbial community variation (*p* < 0.01), followed by TK, pH, and vector angle (VA) (*p* < 0.05). In addition, PLFAs, EEA, and MC were among the top contributors with statistically significant importance, while SOC, TN, TP, and AP also played notable roles, albeit with relatively lower influence.

**Figure 6:**
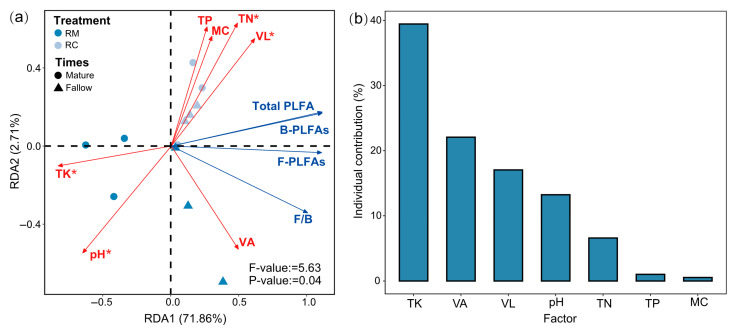
(**a**) Redundancy analysis (RDA) of soil microbial communities and environmental factors; (**b**) Hierarchical partitioning of key environmental drivers. TK, VL, pH, and TN were significant contributors (*p* < 0.05); VA showed a high but non-significant effect. Significance: * *p* < 0.05.

Redundancy analysis (RDA) revealed that the first two canonical axes, RDA1 and RDA2, explained a combined 74.57% of the total variation in soil microbial community composition under RC (*p* < 0.05; Figure 6a). Among these factors, TK emerged as the most critical driver of microbial community structure, followed by vector length (VL), soil pH, and TN, all of which showed significant contributions (*p* < 0.05; Figure 6b). Although the vector angle (VA) had a relatively high contribution rate, its effect was not statistically significant.

## 3. Discussions

### 3.1. Changes in Soil Physicochemical Properties Under Rice–Crayfish Co-Culture Systems

RC significantly improved soil structure, moisture retention, and organic carbon levels compared to RM, demonstrating clear ecological benefits in our study. Two-way ANOVA showed that both growth stage and treatment had significant effects (*p* < 0.001) on MC, SP, BD, and SOC, with treatment also affecting pH. Significant interaction effects (stage × treatment) for MC, pH, and SOC indicate that the system’s impact varied across rice development stages, reflecting dynamic soil–plant–animal interactions [3,15]. These results highlight the potential of integrating aquaculture into rice farming to enhance soil health and promote sustainable paddy ecosystem management [16].

Throughout all rice growth stages, the RC system markedly improved soil conditions compared to RM. At the tillering stage, RC significantly increased MC, SP, and SOC by 30.2%, 9.6%, and 95.8%, respectively, while decreasing BD. These changes indicate an early enhancement of soil structure and water-holding capacity, consistent with benefits previously reported in rice–aquaculture systems [17,18]. The improvement may be due to crayfish-induced bioturbation, which enhances soil aeration and aggregation [19]. The non-significant difference in soil pH at this stage suggests that pH may be more resilient to short-term biological disturbance or influenced by crop growth conditions. In the jointing and heading stages, the benefits of the RC system became even more pronounced. SOC levels in RC plots nearly tripled relative to RM, alongside significantly greater MC and SP and reduced BD and pH. These trends suggest that crayfish activity and increased root exudation under the co-culture system contribute substantial organic inputs and microbial stimulation, thus enhancing SOC accumulation [20]. The observed decrease in soil pH could be linked to the production of organic acids and higher microbial respiration stimulated by the greater availability of organic substrates [21]. Lower BD under RC may facilitate improved root penetration and water infiltration, contributing further to plant and microbial productivity [22]. By the maturity stage, RC plots continued to show improved MC, SP, and SOC, while maintaining lower BD and pH than RM plots. These persistent improvements suggest that the RC system supports long-term enhancement of soil quality, likely contributing to improved nutrient cycling, microbial activity, and overall soil fertility [23]. Interestingly, even in the fallow stage, after harvest and during minimal biological activity, RC plots retained significantly higher MC, SP, and SOC, along with lower BD. This indicates that the positive effects of the RC system on soil structure and C storage extend beyond the active cropping season. These results are consistent with previous research demonstrating the agronomic and ecological benefits of integrating aquaculture into rice farming. Studies on rice–fish and RC systems have shown improvements in soil physical structure, enhanced carbon sequestration, and increased sustainability of rice agro-ecosystems [24,25,26]. Higher MC and SP improve the soil’s capacity to buffer against drought stress, while lower BD fosters better root development and microbial diversity [27,28]. The accumulation of SOC under RC systems has further implications for long-term soil fertility and C mitigation.

### 3.2. Dynamic Changes in Soil Nutrient Availability and Stoichiometry in Rice–Crayfish Systems

RC significantly enhanced soil nutrient dynamics compared to RM, as evidenced by increased levels of total and available forms of N (TN, AN) and P (TP, AP) across all growth stages. These findings align with previous studies that highlight the role of RC in improving soil fertility and nutrient cycling [22,29]. At the tillering stage, the observed increases in TN and TP under RC suggest an early enhancement of nutrient availability, potentially driven by crayfish-induced bioturbation and organic matter deposition [29]. The pronounced rise in AN and AP at this stage further indicates accelerated N mineralization and P mobilization, which are critical for early crop development. These outcomes support the idea that crayfish activity improves microbial turnover and the decomposition of organic residues [10]. During the jointing and heading stages, the continued elevation of TN, AN, and AP under RC reflects sustained nutrient release and transformation in the rhizosphere, likely stimulated by both crayfish behavior and increased microbial activity [30]. At maturity, the consistently higher levels of TN and AN in RC suggest long-term accumulation of organic matter and efficient nutrient recycling, consistent with findings from similar rice–aquaculture systems [31,32]. The persistent advantage of TP and AP levels implies that the system maintains phosphorus availability even in later growth stages, an important factor for grain filling and yield formation [23]. Even in the fallow period, the RC system retained elevated levels of TN and AN compared to RM. This residual fertility may reduce the need for external fertilizer inputs in the following season and reflect the system’s potential for long-term nutrient sustainability. The durability of these nutrient enhancements supports the broader ecological benefits of RC systems.

However, both TK and AK contents showed significant declines under the RC system. This result may be attributed to the rapid uptake of potassium by both plants and animals. During the jointing and heading stages, rice exhibits a high demand for potassium, while crayfish may also consume soluble potassium through feeding or utilize it during physiological activities. These processes likely accelerate the translocation of potassium into plant and animal biomass, thereby reducing TK and AK concentrations in the soil. Moreover, potassium is a highly mobile nutrient, and the RC system typically maintains a shallow water layer in the paddy field. This hydrological condition increases the risk of leaching losses during irrigation and drainage events. Such leaching may persist from the rice growing season into the fallow period, as evidenced by the consistent and significant decrease in AK throughout the entire cultivation cycle [33,34].

From the perspective of ecological stoichiometry, the RC treatment significantly increased soil C:N and C:P ratios during the early growth stages of rice, while the N:P ratio showed no significant change. This phenomenon may be attributed to the substantial input of C-rich organic materials in the early stages of the RC system, including crayfish feces, residual feed, and decomposed plant residues. These inputs are typically high in C content, whereas the mineralization and release of N and P occur at a relatively slower rate. As a result, incomplete decomposition of organic matter in the short term leads to elevated C:N and C:P ratios. In the mid- to late stages of rice growth, the C:P and N:P ratios under RC treatment continued to increase significantly, whereas the C:N ratio remained relatively stable [35]. This trend suggests that, under sustained plant uptake, intensified crayfish activity, and accelerated microbial metabolism, P was continuously absorbed and transformed, eventually resulting in a relative P deficiency in the soil. The increase in C:P and N:P ratios represents a typical ecological response of microbial communities to P limitation, indicating that while microbial C and N demands are being met, the competition for and dependence on P become more pronounced [36]. Overall, these findings highlight the RC system’s effectiveness in enhancing nutrient availability and retention in paddy soils, thereby improving soil fertility, supporting crop productivity, and promoting sustainable agricultural practices [1].

### 3.3. Rice–Crayfish Co-Culture Enhances Soil Enzyme Activities and Alters Microbial Stoichiometric Constraints

The significant enhancement of soil extracellular enzyme activities under the RC system underscores the vital role of integrated aquaculture in promoting microbial-driven nutrient cycling in paddy ecosystems. Elevated activities of C- and N-hydrolyzing enzymes (GCH and GNH), as observed in Figure 2, indicate increased microbial decomposition of organic matter and enhanced N turnover, likely driven by greater organic inputs from crayfish residues and excreta [37,38]. This aligns with previous studies showing that animal integration into agro-ecosystems stimulates microbial metabolism by enriching labile C substrates [39]. The marked increase in acid phosphatase (ACP) activity under RC suggests improved P mineralization and microbial P acquisition. This is particularly important in flooded paddy soils where P availability is often limited due to sorption and redox dynamics [29].

RC significantly influenced soil enzymatic stoichiometry, as indicated by both VL and VA. The higher VL values under RC across all growth stages (Figure 3a) suggest elevated microbial nutrient demand, which is often associated with intensified microbial metabolic activity and faster organic matter turnover [40]. This enhanced microbial functional response likely results from increased organic inputs and bioturbation due to crayfish activity, which can stimulate microbial growth and enzymatic investment [41].

The consistent increase in VA above 45° under RC (Figure 3b) indicates a microbial shift from N limitation toward P limitation. This pattern contrasts with RM plots, where N limitation was observed during the early tillering stage. Such a shift suggests that RC enhances N availability, possibly through crayfish excreta and residue decomposition, more than it does P, leading to an imbalance in microbial nutrient acquisition [42,43]. This stoichiometric imbalance has critical implications for nutrient management in integrated agro-ecosystems. Although the RC system promotes microbial activity and nutrient cycling, the resulting P limitation may constrain long-term productivity if not properly addressed. Targeted phosphorus supplementation or strategies that enhance P availability, such as P-solubilizing bacteria or biochar, may be necessary to sustain soil fertility and system sustainability [44]. RC significantly intensifies microbial nutrient demand and shifts enzymatic stoichiometric patterns toward P limitation. These findings emphasize the importance of adaptive nutrient management strategies to optimize microbial functioning and maintain ecosystem balance in integrated paddy systems.

### 3.4. Rice–Crayfish Co-Culture Reshapes Soil Microbial Community Composition and Enhances Biomass

The results presented in this study highlight the significant influence of RC on soil microbial communities, as evidenced by changes in microbial phospholipid fatty acid (PLFA) profiles. Our findings show that the rice–shrimp integrated farming model enhances soil microbial biomass and alters the microbial community structure, shifting the balance between fungi and bacteria, as well as between Gram-positive and Gram-negative bacteria. These findings are consistent with previous research that has shown that integrated farming systems, including rice–shrimp cultivation, can positively affect soil microbial communities, enhancing biodiversity and microbial activity [15,20,45].

The observed increase in soil microbial biomass under RC (as indicated by the increased PLFA content) is likely due to improved nutrient cycling and organic matter inputs, which are characteristic of integrated systems [25]. In particular, the addition of organic matter from shrimp excretion and rice residue may provide a more diverse nutrient base, promoting the growth of a wider range of soil microorganisms [46]. The higher microbial biomass in RC could also be attributed to the enhanced microbial growth conditions in the root zone, where the rice plants and shrimp interact to create a dynamic and nutrient-rich environment [10]. Fungi are often more prevalent in environments with high organic matter inputs, and the addition of shrimp manure may contribute to this shift by providing additional nutrients [47]. The observed fungal dominance may also indicate a potential improvement in soil health, as fungi contribute to soil structure and the formation of aggregates, which can enhance water retention and nutrient availability [14].

The G^+^/G^−^ ratio is a key ecological indicator in soil microbial ecology, used to assess microbial community structure, environmental stress response capacity, and carbon substrate utilization preference. Variations in this ratio reflect microbial responses to environmental conditions, land-use practices, and nutrient inputs [48]. Generally, Gram-positive bacteria (G^+^) dominate in stable or resource-limited environments, whereas Gram-negative bacteria (G^−^) are more responsive and tend to flourish in resource-rich and frequently disturbed environments, such as those enriched with crayfish feces, residual feed, and plant root exudates [49]. The observed decrease in the G^+^/G^−^ ratio under the RC system indicates a shift in the microbial community structure from a stability-oriented to a more metabolically active type. This transformation is particularly evident during the rice reproductive stage (maturity), when high levels of organic input stimulate G^−^ proliferation and enhance rapid nutrient turnover, thereby contributing to crop productivity. In contrast, the recovery of the G^+^/G^−^ ratio during the fallow period suggests a degree of microbial structural resilience and system stability. These findings highlight the dynamic adaptability and ecological elasticity of microbial communities under RC [47].

Overall, the results of this study underscore the importance of integrated farming practices in enhancing soil microbial diversity and activity. RC not only boosts microbial biomass but also fosters a more balanced and resilient microbial community, which could lead to improved soil health and increased agricultural productivity. These findings align with growing evidence that integrated systems, which combine aquaculture and agriculture, can contribute to sustainable farming practices by enhancing soil health, reducing the need for chemical inputs, and promoting environmental sustainability [17].

### 3.5. Effects of Soil Environmental Factors on Microbial Community Composition

The findings of this study demonstrate that both biochemical factors (such as PLFAs and extracellular enzyme activities) and physical properties (such as MC and BD) significantly influence microbial community composition in RC. These results are consistent with previous studies highlighting the role of soil physicochemical parameters in structuring microbial assemblages [50]. High correlation coefficients for PLFAs and EEA suggest that microbial biomass and activity are central indicators of microbial community shifts under agricultural management [15,51]. The strong positive associations between microbial composition and soil nutrients (e.g., SOC, TN, TP, AP) reflect the dependency of microbial metabolic function on nutrient availability, a relationship widely observed in managed agro-ecosystems [52]. Interestingly, stoichiometric ratios such as C:N, C:P, and N:P also showed significant positive correlations with microbial communities, reinforcing the idea that ecological stoichiometry governs microbial resource use and community turnover [36]. The negative correlations with soil pH and bulk density align with known inhibitory effects of soil acidification and compaction on microbial diversity and activity [49].

The Random Forest analysis provided further insight into the relative importance of individual variables. The identification of VL as the top predictor suggests that integrated multivariate measures may capture complex gradients of microbial responses better than single parameters. The contributions of TK, pH, and VA also suggest that both chemical stressors and spatial environmental heterogeneity play roles in microbial regulation. The combined influence of moisture content, enzyme activity, and nutrient availability indicates that microbial communities are shaped by the interplay between soil biological processes and abiotic constraints, especially in RC, where ecological interactions are intensified [14]. These results underscore the importance of maintaining balanced soil nutrient profiles and optimal physical conditions to sustain microbial diversity and function in integrated rice aquaculture systems. Long-term monitoring and functional gene profiling would further enhance our understanding of microbial roles in nutrient cycling and environmental resilience.

Redundancy analysis (RDA) in this study revealed that a substantial proportion (74.57%) of the variation in soil microbial community composition under RC was explained by selected environmental variables, underscoring the strong influence of abiotic factors on microbial ecology in agro-ecosystems. The prominent role of TK as the primary driver aligns with prior research demonstrating K’s critical role in microbial metabolism and community structuring [53]. K not only acts as an essential macronutrient for microbial enzymatic activities but also influences soil cation exchange capacity and microbial habitat quality [54].

Soil physicochemical properties, notably pH and VL, also significantly shaped microbial communities. Soil pH has long been recognized as a key variable governing microbial diversity and function by affecting nutrient availability and enzyme activities [55]. The importance of VL, which reflects soil structural characteristics, suggests that physical soil properties modulate microbial habitats by influencing aeration, moisture retention, and root–microbe interactions [56]. These factors collectively create a heterogeneous microenvironment critical for microbial niche differentiation. Total nitrogen (TN) was another significant driver, consistent with findings that N availability strongly influences microbial community composition, especially in N-sensitive functional groups involved in nutrient cycling [57]. The integrated effect of nutrient elements (TK, TN) and physicochemical parameters (pH, VL) highlights the multifaceted controls over microbial dynamics in RC, where both biotic interactions and environmental filters interplay. Although vector angle (VA) showed a relatively high contribution to community variation, its lack of statistical significance may indicate a secondary or context-dependent role in microbial structuring. This result suggests that certain soil physical characteristics may interact complexly or indirectly with microbial communities, warranting further investigation using more refined spatial or temporal scales. Overall, these findings underscore the necessity of managing soil nutrient and structural conditions in RC to sustain beneficial microbial communities that can enhance soil health, nutrient cycling, and agro-ecosystem productivity.

## 4. Materials and Methods

### 4.1. Site Description

**Figure 7:**
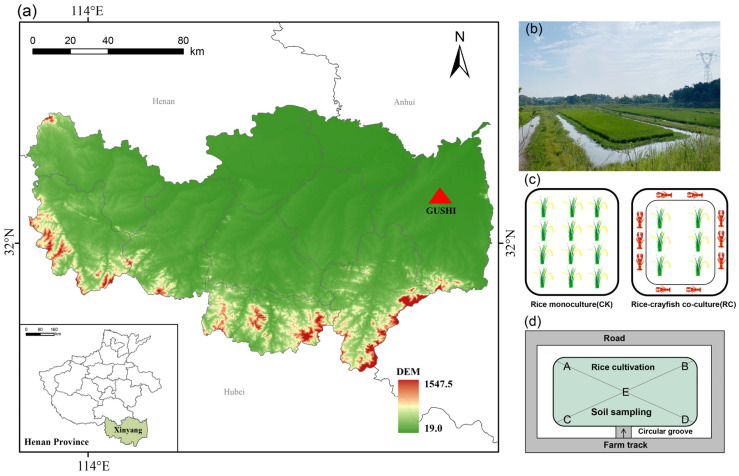
Location and experimental design of the study area. Note: Panel (**a**) shows the map of China, indicating the location of the study site. Panel (**b**) presents a photograph of the rice–crayfish integrated farming system in paddy fields. Panel (**c**) illustrates the two-level experimental design, which includes control plots (rice only) and treatment plots (rice + crayfish). Panel (**d**) displays the X-shaped sampling method used for data collection.

The experiment was conducted in 2023 and 2024 at the Rice–Fish Integrated Farming Demonstration Base in Gushi County, Xinyang City (32°16′ N, 115°79′ E). The experimental site consisted of six plots, each with an area of approximately 0.1 or 0.2 hectares. All selected plots had been under integrated rice–crayfish cultivation for over five years. This region is located in southern Henan Province, characterized by higher elevations in the south and lower elevations in the north. It lies in a transitional zone between subtropical and warm temperate climates. The area experiences a distinct monsoon climate, with a frost-free period of 220–230 days, an annual average temperature of 15.3–15.8 °C, and abundant rainfall ranging from 993 to 1294 mm per year. In southern Henan, a total of 11 national-level and 23 provincial-level rice–aquaculture integrated farming demonstration zones have been established, promoting the certification of green and organic aquatic and rice products. Under this integrated model, the average rice yield is approximately 6150–6750 kg/ha, while the average yield of crayfish (or fish) is around 1050 kg/ha. The average output value reaches approximately 74,000 USD/ha, with an estimated net profit of about 2900 USD/ha for the rice–crayfish system (Figure 7).

### 4.2. Experimental Design

The field experiment was conducted from June 2023 to June 2024 at a rice–aquaculture demonstration base in Gushi County, Xinyang City, China. The experiment consisted of two phases: the rice growing season (June to October 2023) and the fallow period (prior to rice transplanting in early June 2024). All fields were modified with peripheral ring ditches and raised planting beds. Rice was cultivated on the raised beds, while crayfish were raised in the surrounding ditches.

A single-factor completely randomized design (CRD) was used with two treatments: (1) RM as the control and (2) RC as the treatment. Each treatment was replicated three times. In the RC system, rice and crayfish were cultivated simultaneously. During the rice season, crayfish moved freely within the paddy, and during the fallow period, they were confined to the ring ditches. Crayfish juveniles were restocked in March based on population density and harvested in April–May, and rice transplanting took place from late May to early June. Water levels were adjusted throughout the season according to management needs. In contrast, the RM system involved only rice cultivation with no crayfish. All plots used the same irrigation water source (a tributary of the upper Huai River) and had similar soil properties (loam). Pesticides were not used in the RC system; only minimal herbicides (e.g., pyribenzoxim, bensulfuron-methyl, clopyralid) were applied when needed, typically in late May to early June. Conventional pest control was used in RM plots depending on field conditions, mainly from late May to early July.

### 4.3. Sample Collection

Soil and water samples were collected at the end of each period (post-harvest and pre-transplanting) from each plot. Sampling followed an X-shaped pattern (Figure 7d) within each plot to ensure spatial representation. Composite samples were obtained by mixing five subsamples per plot. Soil samples were taken from the top 0–20 cm layer using a 40 mm diameter soil auger. Plant debris, roots, stones, and other impurities were removed. Each composite soil sample was split into two portions: one was air-dried for physicochemical analysis, and the other was placed in sterile sealed containers, transported in liquid nitrogen or on dry ice, and stored at –80 °C for microbial and extracellular enzyme activity analysis. Soil sampling was conducted during four key rice growth stages and the fallow period. The specific sampling dates were as follows: tillering stage (11 July 2023), jointing stage (18 August 2023), heading stage (24 August 2023), maturity stage (22 September 2023), and fallow period (8 June 2024). Water samples were collected from both the ring ditches and paddy surfaces (if applicable), stored in clean containers, and transported to the laboratory under chilled conditions for quality assessment. Importantly, herbicides were applied only prior to the sampling dates. No plant protection products were used during or after sampling, ensuring that the collected samples reflected the actual post-treatment field conditions for accurate microbial and chemical assessments.

### 4.4. Soil Measurement and Analysis

Soil bulk density and total porosity were determined using the ring knife method. Fresh soil samples were oven-dried at 105 °C until a constant weight was reached to measure SM. Soil pH was measured in a soil–water suspension (1:2.5, *w*/*v*) using a pH meter (Model PHS-3C, Shanghai Instrument Scientific Co., Ltd. Shanghai, China). Soil organic matter (SOM) and SOC were quantified via the potassium dichromate (K_2_Cr_2_O_7_) oxidation method. Total nitrogen (TN) was determined by the Kjeldahl digestion method, while alkaline hydrolyzable nitrogen was measured using the alkali diffusion method. Total phosphorus (TP) was analyzed by the molybdenum–antimony anti-colorimetric method, and AP was extracted with double acid and measured using a microplate reader. Total potassium (TK) and AK were measured by flow injection analysis (SEAL-AA3 system, SEAL Analytical GmbH, Norderstedt, Schleswig-Holstein, Germany).

Extracellular enzyme activities related to nutrient acquisition were assayed using commercial kits (Solaibao Technology Co., Ltd. Beijing, China). These included N acquisition enzymes β-N-acetylglucosaminidase (NAG) and L-leucine aminopeptidase (LAP), C acquisition enzymes cellobiohydrolase (CBH) and β-1,4-glucosidase (BG), and P acquisition enzyme acid phosphatase (ACP). Enzyme activities were expressed as nanomoles of substrate released per hour per gram of dry soil (nmol h^−1^ g^−1^).

The geometric mean of five hydrolytic enzyme activities (GH) was calculated to represent the overall nutrient acquisition capacity of soil microbial communities (Equation (1)). Additionally, geometric means of C-related enzymes (GCH) and N-related enzymes (GNH) were computed to estimate microbial carbon and N acquisition capabilities, respectively (Equations (2) and (3)) [58,59].

The geometric mean of the five hydrolytic enzymes (GH) was calculated as follows:

(1) $$GH=\sqrt[{5}]{BG\times CBH\times NAG\times LAP\times ACP}$$

The geometric mean of carbon-acquiring enzymes (GCH) was calculated as:

(2) $$GCH=\sqrt[{2}]{BG\times CBH}$$

The geometric mean of nitrogen-acquiring enzymes (GNH) was calculated as:

(3) $$NH=\sqrt[{2}]{NAG\times LAP}$$

where BG is β-1,4-glucosidase, CBH is cellobiohydrolase, NAG is β-N acetylglucosaminidase, LAP is L-leucine aminopeptidase, and ACP is acid phosphatase.

Based on the stoichiometric characteristics of enzyme activities, the VL reflects the relative degree of microbial carbon limitation. The longer vector lengths indicate stronger C limitation. The VA represents the relative limitation by N or P. Specifically, a vector angle less than 45° indicates a greater N limitation, whereas an angle greater than 45° suggests a greater P limitation. The farther the angle deviates from 45°, the stronger the nutrient limitation (either N or P). The formulas for calculating vector length and vector angle are as follows:

(4) $$Vector\;length=\sqrt{{\left(\frac{BG+CBH}{BG+CBH+NAG+LAP}\right)}^{2}+({\frac{BG+CBH}{BG+CBH+ACP})}^{2}}$$

(5) $$Vector\;angle=Degrees(ATAN2((\frac{BG+CBH}{BG+CBH+ACP}),(\frac{BG+CBH}{BG+CBH+NAG+LAP})))$$

### 4.5. Soil Microbial Phospholipid Fatty Acid (PLFA) Analysis

Soil microbial biomass was assessed using the phospholipid fatty acid (PLFA) method, following the procedures described by Bossio and Scow [60]. Phospholipids were extracted from fresh soil samples using a mixture of chloroform, methanol, and phosphate buffer (1:2:0.8 *v*/*v*/*v*). The phospholipid fraction was then separated using a silica gel column. After separation, the phospholipids were subjected to methylation with a methanol:toluene solution (1:1 *v*/*v*) and KOH in methanol to form fatty acid methyl esters (FAMEs). These FAMEs were then extracted with n-hexane to obtain purified samples. PLFAs were quantified using a gas chromatograph (Trace 1300E, Thermo Fisher Scientific, Waltham, MA, USA), with nonadecanoic acid methyl ester (19:0) used as an internal standard. PLFA components were identified and analyzed using the MIDI Sherlock Microbial Identification System. The results were expressed as micrograms of PLFA per gram of dry soil (μg/g).

### 4.6. Data Analysis

All experimental data were initially organized and preprocessed using Microsoft Excel 2024. Normality of the data was assessed using the Shapiro–Wilk test (α ≥ 0.05), and descriptive statistics, including mean, standard deviation, and standard error, were computed using IBM SPSS Statistics 27.0. A two-way analysis of variance (ANOVA) was performed to evaluate the effects of different treatments on soil physicochemical properties, extracellular enzyme activities, vector model parameters, and microbial biomass, with statistical significance determined at *p* < 0.05. Graphical visualizations of soil properties, enzyme activity, and microbial indicators were created using GraphPad Prism 10.0. The relationships between soil extracellular enzyme activities, microbial biomass, and environmental factors were examined using Mantel tests in R version 4.4.2. A higher correlation coefficient and a lower *p*-value indicated a stronger influence of environmental factors on microbial community dynamics. To further explore the underlying relationships, a Random Forest model (implemented via the Random Forest package in R) was used to assess the nonlinear associations between soil environmental variables and microbial biomass and to rank the relative importance of predictors. In addition, Redundancy Analysis (RDA) was conducted to quantify the direct contributions of soil environmental factors to variations in microbial community composition. Future research should explore the vertical distribution of soil nutrients and microbial communities, particularly in the 20–40 cm soil layer, to better understand the deeper impacts of the rice–crayfish co-culture system.

## 5. Conclusions

This study demonstrates that the rice–crayfish (RC) system can significantly enhance soil physicochemical properties, nutrient availability, and microbial communities in paddy fields compared to traditional RM. Improvements in soil moisture, organic carbon, and nutrient content under RC conditions contribute to better soil fertility and a more favorable environment for plant and microbial growth. Enhanced microbial functional activity and altered community composition under the RC system suggest increased biodiversity and ecosystem resilience, which are essential for sustainable nutrient cycling and long-term agro-ecosystem stability. Multivariate analyses highlight the integrated effects of soil biochemical and physical properties on microbial dynamics, supporting the value of RC as a holistic and sustainable farming strategy. However, this study is limited to surface soil (0–20 cm) and lacks direct measurements of nutrient leaching and potassium removal via crayfish harvesting. Future research should address these gaps by exploring vertical soil profiles, long-term nutrient dynamics, and the coupling between soil health, microbial communities, and economic yield. Such efforts will provide a more comprehensive understanding of the ecological and agronomic benefits of rice–crayfish co-culture systems.

## Figures and Tables

**Table 1:** Effects of a rice–crayfish co-culture system on soil physicochemical properties.

Stage	Treatment	MC	SP	BD	pH	SOC
Tillering	RM	0.53 ± 0.02 b	56.66 ± 0.43 b	1.15 ± 0.01 a	6.45 ± 0.24 a	3.59 ± 0.17 b
	RC	0.69 ± 0.01 a	62.08 ± 0.89 a	1.01 ± 0.02 b	6.62 ± 0.05 a	7.03 ± 0.59 a
Jointing	RM	0.47 ± 0.01 b	52.88 ± 1.32 b	1.25 ± 0.03 a	6.8 ± 0.07 a	3.91 ± 0.06 b
	RC	0.55 ± 0.01 a	61.08 ± 1.22 a	0.99 ± 0.05 b	5.87 ± 0.09 b	11.72 ± 0.13 a
Heading	RM	0.47 ± 0.03 b	54.23 ± 0.48 b	1.21 ± 0.01 a	6.92 ± 0.06 a	4.09 ± 0.36 b
	RC	0.53 ± 0.01 a	58.49 ± 0.87 a	1.1 ± 0.02 a	5.54 ± 0.28 b	13.61 ± 0.45 a
Mature	RM	0.37 ± 0.01 b	59.17 ± 1.1 b	1.07 ± 0.04 a	6.94 ± 0.02 a	4.17 ± 0.42 b
	RC	0.52 ± 0.03 a	64.27 ± 0.64 a	0.91 ± 0.04 b	6.04 ± 0.1 b	12.19 ± 0.64 a
Fallow	RM	0.31 ± 0.01 b	49.43 ± 0.77 b	1.34 ± 0.02 a	6.44 ± 0.15 a	4.32 ± 0.23 b
	RC	0.43 ± 0.01 a	56.16 ± 0.38 a	1.16 ± 0.01 b	6.03 ± 0.05 a	11.26 ± 0.26 a
ANOVA						
Stage, S		***	***	***	ns	***
Treatment, T		***	***	***	***	***
S × T		***	ns	ns	***	***

Note: Values are presented as mean ± standard error (*n* = 3). Different letters (a, b) within the same growth stage indicate significant differences between treatments at *p* < 0.05. Significance levels: *** *p* < 0.001 (highly significant), and ns: not significant. Abbreviations: MC: soil moisture content; SP: soil porosity; BD: bulk density; pH: soil pH; SOC: soil organic carbon.

## Data Availability

The datasets generated for this study are available on request to the corresponding author.
